# Memorable Food: Fighting Age-Related Neurodegeneration by Precision Nutrition

**DOI:** 10.3389/fnut.2021.688086

**Published:** 2021-08-05

**Authors:** Maja Milošević, Aleksandra Arsić, Zorica Cvetković, Vesna Vučić

**Affiliations:** ^1^Department of Neuroendocrinology, Institute for Medical Research, University of Belgrade, Belgrade, Serbia; ^2^Department of Nutritional Biochemistry and Dietology, Centre of Research Excellence in Nutrition and Metabolism, Institute for Medical Research, University of Belgrade, Belgrade, Serbia; ^3^Department of Hematology, Clinical Hospital Center Zemun, Belgrade, Serbia; ^4^Faculty of Medicine, University of Belgrade, Belgrade, Serbia

**Keywords:** neurodegenerative diseases (MeSH), epigenetics (DNA methylation, histone modifications), gut microbiota, aging, precise nutrition

## Abstract

Healthcare systems worldwide are seriously challenged by a rising prevalence of neurodegenerative diseases (NDDs), which mostly, but not exclusively, affect the ever-growing population of the elderly. The most known neurodegenerative diseases are Alzheimer's (AD) and Parkinson's disease, multiple sclerosis, and amyotrophic lateral sclerosis, but some viral infections of the brain and traumatic brain injury may also cause NDD. Typical for NDD are the malfunctioning of neurons and their irreversible loss, which often progress irreversibly to dementia and ultimately to death. Numerous factors are involved in the pathogenesis of NDD: genetic variability, epigenetic changes, extent of oxidative/nitrosative stress, mitochondrial dysfunction, and DNA damage. The complex interplay of all the above-mentioned factors may be a fingerprint of neurodegeneration, with different diseases being affected to different extents by particular factors. There is a voluminous body of evidence showing the benefits of regular exercise to brain health and cognitive functions. Moreover, the importance of a healthy diet, balanced in macro- and micro-nutrients, in preventing neurodegeneration and slowing down a progression to full-blown disease is evident. Individuals affected by NDD almost inevitably have low-grade inflammation and anomalies in lipid metabolism. Metabolic and lipid profiles in NDD can be improved by the Mediterranean diet. Many studies have associated the Mediterranean diet with a decreased risk of dementia and AD, but a cause-and-effect relationship has not been deduced. Studies with caloric restriction showed neuroprotective effects in animal models, but the results in humans are inconsistent. The pathologies of NDD are complex and there is a great inter-individual (epi)genetic variance within any population. Furthermore, the gut microbiome, being deeply involved in nutrient uptake and lipid metabolism, also represents a pillar of the gut microbiome–brain axis and is linked with the pathogenesis of NDD. Numerous studies on the role of different micronutrients (omega-3 fatty acids, bioactive polyphenols from fruit and medicinal plants) in the prevention, prediction, and treatment of NDD have been conducted, but we are still far away from a personalized diet plan for individual NDD patients. For this to be realized, large-scale cohorts that would include the precise monitoring of food intake, mapping of genetic variants, epigenetic data, microbiome studies, and metabolome, lipidome, and transcriptome data are needed.

## Introduction

Because of increasing life expectancy and decreasing birth rates, the world's population aged 60 years and older is expected to total 2 billion by the year 2050, and 80% of the elderly will be living in low- and middle-income countries, according to a World Health Organization report (1). Healthcare systems all over the world are seriously challenged by a rising prevalence of disabling chronic diseases, including cancer, cardiovascular disease and neurodegenerative disease (NDD), which affect mostly, but not exclusively, the ever-growing population of the elderly. Gradual and progressive severe damage in neuronal cells lead to severe memory and behavioral impairment (dementia) and loss of movement control (ataxia and paralysis) making NDD the major cause of disability and morbidity among older people worldwide. The most common NDDs are Alzheimer's disease (AD), Parkinson's disease (PD), multiple sclerosis (MS), and amyotrophic lateral sclerosis (ALS), but some viral infections of the brain and traumatic brain injury may also cause NDD. Moreover, a significant proportion of the older population is affected by “age-related cognitive decline,” which is independent of dementia and has an incidence 70% higher than dementia alone (2). These patients experience increasing deficits in daily living activities, productivity losses, and subsequently need constant and long-term care, which is connected with overwhelming economic and societal cost (3, 4). Thus, healthy aging and the prevention of neurodegeneration is emerging as a global ultimate goal.

Several cellular and molecular determinants of aging have been identified so far, including loss of protein homeostasis (proteostasis), stem-cell exhaustion, mitochondrial dysfunction, genomic instability, epigenetic alterations, telomere attrition, cellular senescence (i.e., permanent proliferation arrest), deregulated nutrient sensing, altered intracellular signaling, and synaptic dysfunction (5–7). The complex interplay of all the above-mentioned factors is a fingerprint of neurodegeneration, with different diseases being affected to different extents by specific factors.

The phenotypes of aging can be modified to increase longevity and to prevent or to delay the onset and/or to ameliorate the clinical course of neurodegeneration. Besides drugs approved by the Food and Drug Administration (FDA), such as acetylcholine esterase and levodopa for PD, that ameliorate the symptoms and slow down the progression of NDD, many other medications, such as rapamycin, senolytics, metformin, acarbose, spermidine, and NAD^+^ enhancers, may improve the quality of life by preserving functional capacity and decreasing disease burden in the elderly and are currently being intensively investigated (8). There is increasing evidence that regular exercise and healthier dietary patterns, balanced in macro- and micro-nutrients, can also have beneficial effects on brain health and cognitive functions by modifying the above-mentioned age-related molecular determinants.

## Role of Diet in Neurodegeneration

The important role of deleterious dietary behavior (overfeeding, a high caloric/low dietary fiber diet, or the low consumption of antioxidant nutrients), environmental factors (smoking, alcohol, stress, drugs, and exposure to pesticides), and a sedentary lifestyle throughout the entire life span, including early life, in the development of neurodegeneration is now well-recognized (9). The unbalanced diet during pre-conception, pregnancy, and the first 2 years of life is associated with the inheritance of epigenetic alterations that promote neurodegeneration and are transmissible to offspring and to subsequent generations (10). In addition, an unhealthy diet may alter the gut microbiota, including the neonate's microbiota *via* breastfeeding as a result of the mother's diet, and promote the development of NDD (11). Generally, the consumption of diets rich in antioxidants and anti-inflammatory components and reduced caloric intake may lower age-related cognitive decline and the risk of NDD (12). Fruits, vegetables, beverages, green tea, coffee, spices, nuts, and cereal products are major sources of plant-derived antioxidants—polyphenols (phenolic acids, flavonoids, anthocyanins, lignans, and stilbenes), carotenoids (xanthophylls and carotenes), and vitamins (vitamins E and C)—and their beneficial effect in NDD has been previously reviewed (13). *In vitro* and *in vivo* studies have proposed the neuroprotective properties of vitamin D (14), B vitamins (B12, B6 and riboflavin) (15), vitamin K (16), and trace elements such as selenium, copper, magnesium, iron, lithium, and zinc (17) on neurocognitive disorders, mitochondrial dysfunction, immune dysfunction, inflammatory conditions, cognition, and memory. The neuroprotective role of polyunsaturated fatty acids (PUFAs) and their positive effect in prevention and treatment in NDD is documented in nutritional epidemiology studies, prospective population-based surveys and clinical trials (18, 19). Metabolic and lipid profiles in NDD can be improved by healthy dietary patterns, such as the Mediterranean diet.

The traditional diet consumed in Mediterranean countries is characterized by a high intake of vegetables, legumes, fruits, nuts, and wholegrains, a moderate intake of fish, poultry, and red wine (with meals), and a low intake of red and processed meats, with olive oil used as the main fat source; as a whole the diet has a positive effect on diabetes, cardiovascular disease, and many other chronic conditions (20), as well as aging (5). The nutritional value of this diet implies the consumption of antioxidants, vitamins, trace elements, and PUFAs, in particular ω-3 PUFA. It has been reported that an increased adherence to the Mediterranean diet over a longer period (above 4–6 years) contributes to neuronal integrity (increases cortical thickness and brain volume, slows down the rate of hippocampal atrophy and amyloid accumulation, and improves structural connectivity), as well as cognition, memory, and executive function (21).

As such findings are fragmented and sometimes inconsistent, the optimal daily doses of particular macro- and micro-nutrients in preventing, slowing, and reversing neurodegeneration are still to be established in different population subgroups. Precision nutrition and precision medicine, based on the phenotype of aging, food preferences, clinical history, and lifestyle patterns, are becoming important issues with regard to neurodegeneration. The mechanisms by which neurodegeneration can be fought through “memorable” food, with a focus on epigenetic mediation, intervening in the gut microbiota's composition, the reversal of low-grade inflammation and anomalies in lipid metabolism, and caloric restriction, are further discussed.

## Epigenetics in Neurodegeneration

An increasing body of evidence suggests the role of epigenetic modifications in the development of NDD. Epigenetics encompasses a wide range of stable inheritable and reversible modifications that result in changes in gene expression and function, without affecting the DNA sequence (22). The epigenome constantly changes during the lifespan of an individual. Some of the epigenetic modifications are intrinsically programmed and essential for normal development, growth, and differentiation. The others result in inappropriate epigenetic reprogramming. Although epigenetic modifications are quite stable, they can be modulated by physiological and pathological conditions as well as by the environment (23).

These modifications typically arise owing to DNA methylation or hydroxymethylation, histone post-translational modifications, synthesis of microRNA (miRNAs) and long non-coding RNAs (lncRNAs), and changes in nucleosome positioning, thereby regulating patterns of gene expression. In normal cells these changes are well-balanced and affected by genetic factors (24), environmental factors (25), and stochastic (undetermined) factors. The influence of hereditary factors in epigenetic changes over time has been shown in studies of monozygotic twins (26), dizygotic twins (27), as well as by the familial clustering of DNA methylation found in longitudinal studies (28). In addition, the epigenetic process can also be affected by nutritional and environmental factors and thereby be dynamically changed during the lifespan of an organism (29, 30). Although methylation was originally thought to serve as a stable mark of gene silencing, nowadays it is known that these changes in DNA methylation can be both rapid and reversible (31). Several studies have shown that nutrition- and environment-induced epigenetic modifications can occur at any stage of life, from the *in utero* period throughout adult life and aging, and they can be maintained through multiple offspring generations (32). Epigenetic changes may lead to mutations, and, conversely, mutations are frequently observed in genes that modify the epigenome (33).

## Dna Methylation

Among all epigenetics processes, the most common and investigated is the methylation of DNA.

**Figure 1:**
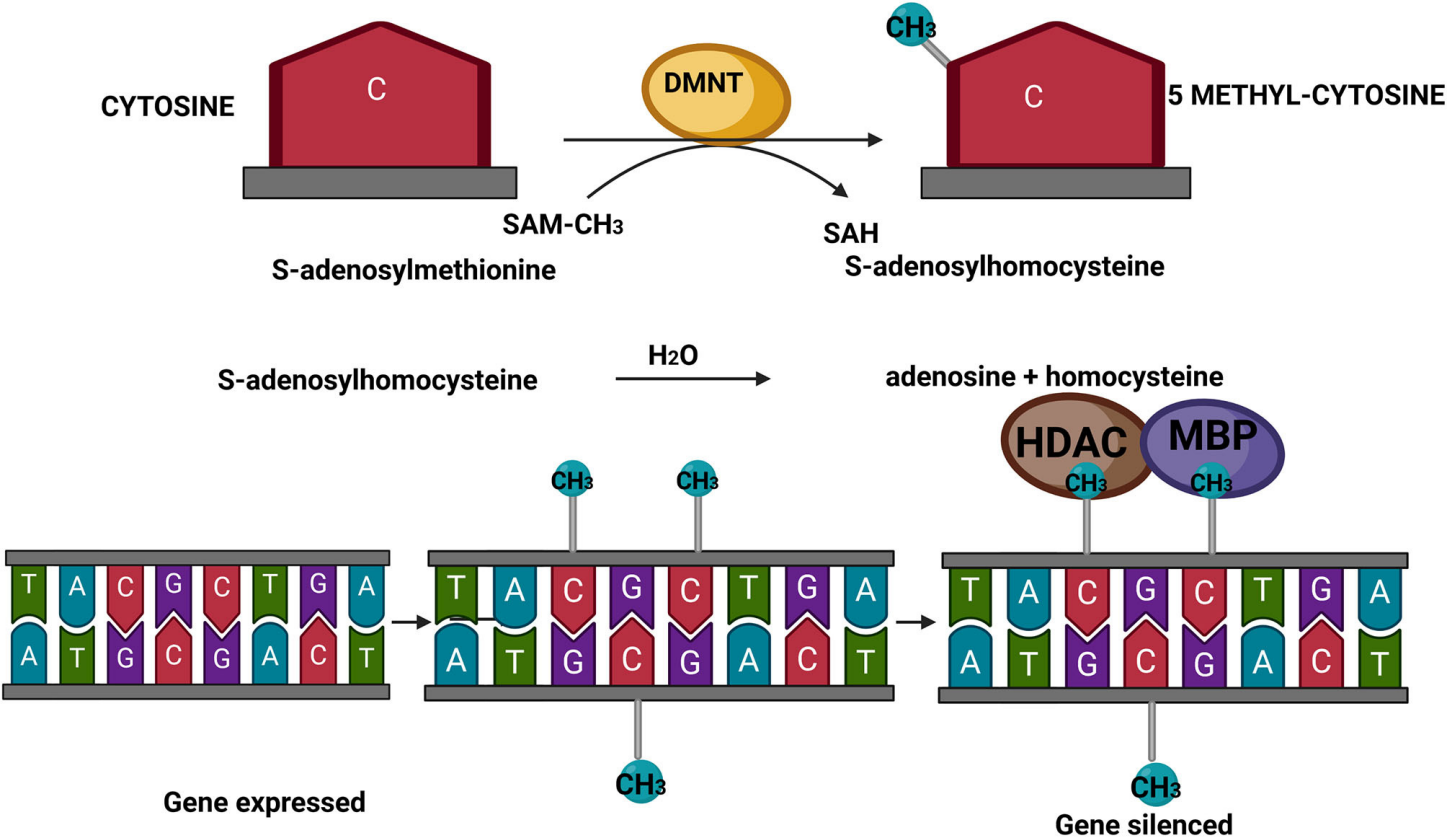
Schematic presentation of DNA methylation. SAM, S-adenosyl methionine; SAH, S-adenosylhomocysteine; DNMTs, DNA methyltransferases; MBPs, methyl-binding proteins; HDACs, histone deacetylases. Created with BioRender.com.

As presented in Figure 1, the mechanism of DNA methylation implies the presence of S-adenosyl methionine (SAM) as the universal methyl donor for DNA and histone proteins. SAM donates the methyl group to the C5 atom of the cytosine moieties followed by guanines, the so-called CpG dinucleotide. This conversion involves the action of DNA methyltransferases (DNMTs). In turn, SAM becomes S-adenosylhomocysteine (SAH), which acts as a competitive inhibitor of methyltransferases, including DNMTs (34) (Figure 1). The inhibition is limited as SAH is rapidly hydrolyzed to adenosine and homocysteine. The main role of DNA methylation is to reduce or impair the binding of transcription factors to the regulatory regions, i.e., promoters of genes. Moreover, DNA methylation results in the recruitment of methyl-binding proteins (MBPs) and histone deacetylases (HDACs) at the methylated site of promoter regions, thereby repressing the expression of genes. In line with this, actively transcribed genes have hypomethylated promoters, whereas hypermethylated promoters are normally associated with silenced, non-expressed genes (35).

There is growing evidence that altered DNA methylation contributes to the occurrence of several diseases (36). Different types of cancers and allergic, immunological, and inflammatory diseases are closely associated with the epigenetic changes of DNA (37–39). Besides tumors, the main class of diseases associated with epigenetic modifications is neurodegenerative disease (40). The association between DNA methylation and NDD is confirmed in Parkinson's disease (41), Alzheimer's disease (42), amyotrophic lateral sclerosis (43), and multiple sclerosis (44). In these diseases, some of the genes are hypermethylated while others are hypomethylated. Thus, in AD, several genes are hypermethylated (*APOE, MTHFR, MAPT, SORB3*), while others included in Aβ peptide production (*PSEN1, APP, PP2A, CREB5, S100A2, BACE*) are hypomethylated (45). Studies have indicated a positive effect of SAM donors on cognitive function and AD in animals and humans through downregulation of the *PSEN1* gene (46, 47). Thus, in a few animal studies conducted in the mice model for AD, supplementation with SAM as the methyl donor modulates the methylation in *PSEN1*, which leads to not only restoring the methylation potential but also losing the symptoms linked with AD (48, 49).

Besides DNA methylation, histone acetylation is a reversible epigenetic modification controlled by histone acetyltransferases and deacetylases. The acetylation of lysine residues on histones decreases the electrostatic attraction between the histones and the DNA backbone and consequently increases transcription. In NDD, histone acetylation homeostasis is markedly impaired, shifting toward hypoacetylation. Enhanced histone acetylation, promoted by HDAC inhibitors, improves learning and memory and has a neuroprotective effect (50).

Many different environmental stressors, such as diet, pollutants, pesticides, chemical species, drugs, physical exercise, and stress, are known to be causative of epigenetic changes (35). They can switch on/off corresponding genes either by direct interaction with DNA, RNA, or chromatin receptors or indirectly using various enzymes or other epigenomic-associated pathways (51–53).

## Nutriepigenomics

Many nutrients from food interact with the DNA, and these interactions are studied by nutriepigenomics. Nutrients affect human health *via* epigenetics without alterations in the DNA sequence in two ways—by promoting epigenetic modifications and by reversing the previous or inherited changes. With regard to food, there are differences between synthetic xenobiotics (bisphenol and glyphosate), which are consumed with food and have an epigenomic effect, and diet and its many micro- and macro-nutrients that have also demonstrated epigenetic effects in *in vitro* and *in vivo* studies, as well as in clinical trials. Toxic xenobiotics may induce DNA methylation in different ways: directly *via* the inhibition of DNA methyltransferases, which leads to hypomethylation, or by subtracting methyl groups from the physiological reactions in which they are included (53).

On the other hand, some nutrients can not only prevent the hypermethylation of DNA but also promote demethylation and the reversal of genes silenced by previous DNA methylation. Thus, molecules such as B vitamins act as methyl donors and might avert the loss of DNA methylation induced by air pollution or some other cause (54). In addition, some bioactive compounds can reverse the epigenome dysregulation induced by bisphenol A (55), while dietary folic acid supplementation can prevent the adverse effects caused by heavy metals (56).

In general, vegetables and fruits and their active molecules have epigenetics potential, and they can modulate DNA methylation. To date, many bioactive components, such as lycopene, hesperidin, phloretin, genistein, coumaric acid, caffeic acid, isothiocyanates, and epigallocatechin gallate, have been identified as those with strong epigenetic potential (Table 1). These molecules exert different effects on the levels of DNA methylation. While some of them show hypermethylating activity, there are those with hypomethylating effects. Thus, tea flavonoids, such as catechin, epicatechin, epicatechin 3-gallate, epigallocatechin, and quercetin, or parsley's apigenin inhibit DNA methylation, leading to demethylation and the reactivation of genes previously silenced by methylation (57). Many of them inhibit DNA methylation in a direct manner by forming hydrogen bonds between different residues in the active site of DNMT (34) or indirectly by decreasing the level of SAM and increasing the levels of both SAH and homocysteine, which subsequently leads to the inhibition of DNA methylation (58). Similarly, resveratrol, found in grapes and red wine and also in peanuts, mulberries, and cranberries, modulates DNA methylation and histone modification *via* the inhibition of DNMTs and histone deacetylase activities (59). In addition, several studies indicate that caffeic acid, present in coffee and barley grain (60), and polyphenols from olive oil can induce the inhibition of DNA methylation (61). In addition, some bioactive molecules, such as curcumin, have both hyper- and hypo-methylating effects on different genes in different cancers, with the same outcome on the tumor (62–64). They can activate some tumor suppressor genes and inactivate oncogenes. Although the protective effects of Ginkgo biloba extract and its flavonoid kaempferol ellagitannin have been widely investigated in AD, its role in the epigenetic alterations related to AD pathogenesis has not been fully finalized. Namely, the inhibition of HDAC activity by kaempferol is confirmed in human-derived hepatoma and colon cancer cells but not in NDD (65).

**Table 1:** Bioactive compounds from food with effects on NDD.

**Food**	**Compound**	**Action**	**Role in NDD**	**References**
Nuts	Ellagic acids	Inhibit HATs; Activate HDACs	Reverse brain atrophy in AD	(123, 124)
Fish oil	ω-3 PUFA	Activates or inhibits DNMTs in different cells	Prevents age-associated cognitive decline	(18, 68, 69)
Olive oil	Gallic acid	Inhibits HATs	Potential prevention of AD	(123, 125)
Ginkgo biloba extract	Kaempferol	Inhibits HDACs	Improves cognitive function in patients with mild dementia during long-term administration	(65, 126)
Red wine	Resveratrol	Activates HATs; Inhibits DNMTs and HDACs	Reduces the risk of AD	(59, 115, 127)
Berries	Gallic and ellagic acids	Inhibit HATs; Activate HDACs and DNMTs	Delay the development of age-related cognitive decline	(123, 128)
Tea	Epigallocatechin-3-gallate	Inhibits DNMTs and HATs	Low prevalence of AD	(57, 129, 130)
Turmeric	Curcumin	Activates HDACs; Inhibits HATs, DNMTs, and miRNA	Corrects the dysregulation of several pathways in NDD	(62–64, 131, 132)
		Alters the relative abundances of bacterial species	Gut microbiota produces neuroprotective metabolites from curcumin	(117)
Cocoa	Epicatechin	Inhibits HATs; Activates HDACs; Increases the presence of “healthy” bacteria	Decreases cerebral inflammation	(119, 133)
Pomegranate	Ellagitannins	Inhibits HATs	Protective effects against AD	(122, 123)
Morinda officinalis	Oligosaccharides	Not determined	Regulates the synthesis and secretion of neurotransmitters in rats	(120)
Algae	Oligomannate	Not determined	Inhibits AD progression in AD mouse models	(121)

*AD, Alzheimer's disease; NDD, neurodegenerative disease; HATs, histone acetyltransferases; HDACs, histone deacetylases; HMTs, histone methyltransferases; miRNA, microRNA*.

## Epigenetic Effects of Fatty Acids

Apart from polyphenols, fatty acids may also be involved in epigenetics transformation. Dietary PUFAs play a significant role in regulating the epigenome, especially in modifying DNA methylation (66). On other hand, there is an opposite correlation, i.e., epigenetic processes can be involved in PUFA biosynthesis processes. However, like polyphenols, the epigenetics roles of PUFAs have been mostly investigated in tumor cells, but not in NDD. Thus, Huang et al. demonstrated that treatment with ω-3 PUFA induced decreased tumor incidence and tumor size in a colorectal cancer rat model (67). They showed that there was a close correlation between the anticancer effects of ω-3 PUFA and increased genomic DNA hydroxymethylation, leading to the silencing of some genes. On the other hand, Sarrabi et al. indicated that PUFA treatment caused the decreased methylation of different oncogenes and suggested that PUFAs can alter both DNA methylation and the expression of DNMTs in colorectal cancer cells (68). Similarly, Ceccarelli et al. showed that ω-3 PUFA directly regulates and demethylates DNA in hepatocarcinoma cell lines (69). Thus, dietary supplementation with bioactive compounds and PUFA may lead to better prognoses for diseases that are associated with epigenetics modulation, including NDD.

Epigenetics development and the identification of highly sensitive, specific, and easily accessible epigenetic biomarkers and applying them, along with genetic biomarkers, is a key step toward successful personalized treatment. Besides personal genetic and epigenetic information, other data, including gender, age, gut microbiota, and presence of diseases, should be taken into account for personalizing prevention and treatment (70). These differences among individuals result in different responses to similar treatments and suggest the need for a personalized approach.

## Microbiota and Gut–Brain Axis

Apart from (nutri)epigenetics, the latest research has shown that the gut microbiota affects the brain's physiological, behavioral, and cognitive functions, although the exact mechanisms have not been fully clarified. The intestinal microbiota represents about 10^14^ microbial cells including bacteria, archaea, viruses, fungi, and protozoa populating the gastrointestinal tract and maintaining a symbiotic relationship with the host. Most of the microbial species in the human gut belong to five phyla: *Firmicutes* and *Bacteroidetes* are dominant, whereas *Actinobacteria, Proteobacteria*, and *Verrucomicrobia* represent minor constituents. Dysbiosis, the imbalance in the composition and function of the gut microbiota, has been implicated in the development of chronic diseases, including gastrointestinal, autoimmune, metabolic, and neurodegenerative diseases (71).

Numerous factors may have harmful effects on the microbiome such as diet, food additives, pesticides, antibiotics, and stress. The balance of and symbioses with the gut microbiome that have been established during human evolution and the rapid change of diet in the last 100 years may outpace the time necessary for the adaptation of the digestive system, resulting in increased occurrence of chronic diseases. Ultra-processed food and excessive energy intake are dominating hallmarks of the Western diet. This diet, abundant in saturated fat and refined carbohydrates, negatively impacts on gut microbiome composition and consequently on the immune system and brain health (72). On the other hand, epidemiological data suggest that caloric restriction and dietary intervention using certain macronutrients (fish), micronutrients (B vitamins, vitamins C, E, and D, flavonoids, and omega-3 PUFA), probiotics, and prebiotics may prevent cognitive decline and/or delay age-related neurodegeneration (73). Such a type of diet is the Mediterranean diet.

**Figure 2:**
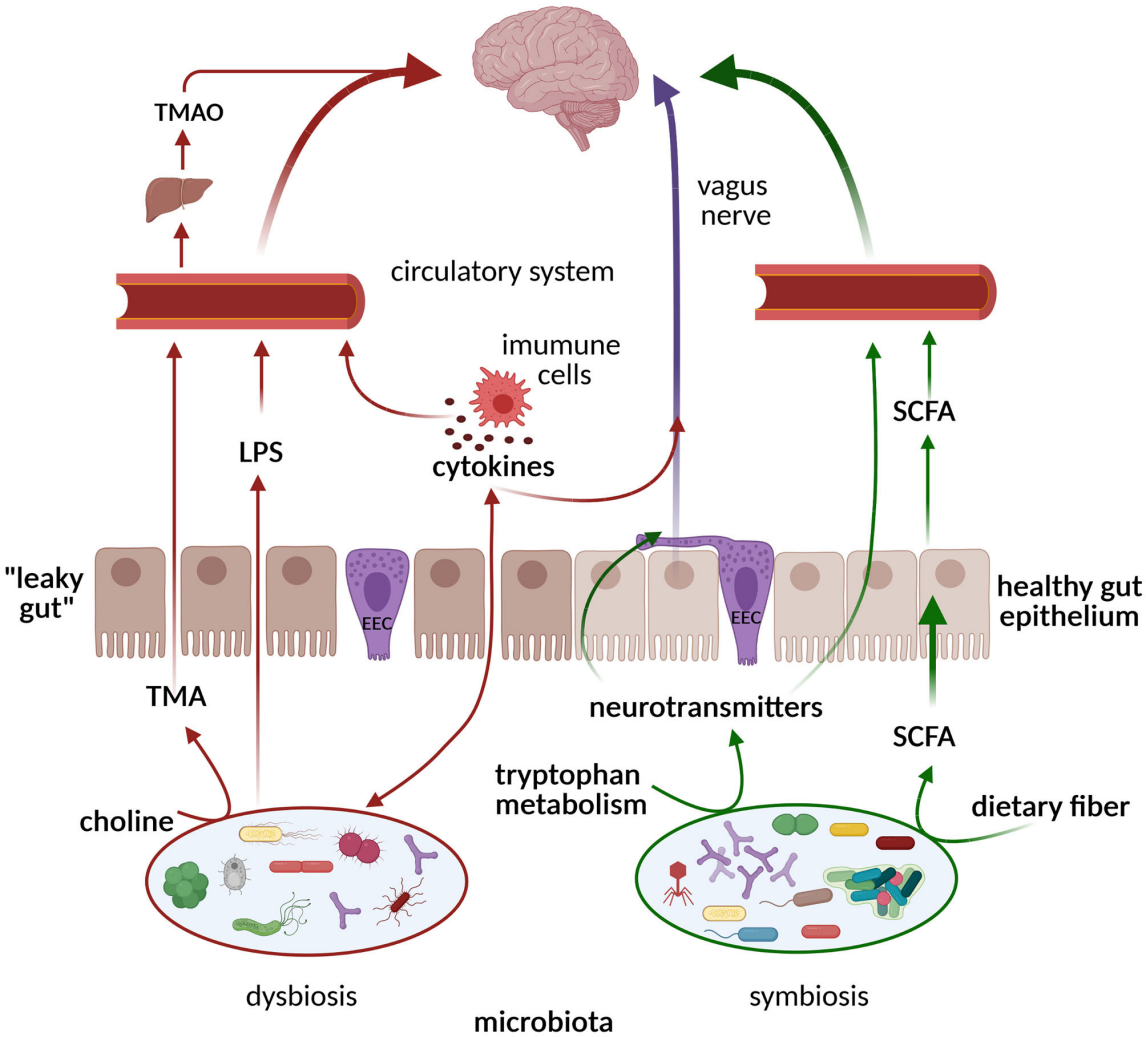
Pathways of communication along the gut microbiome–brain axis. In the state of symbiosis, the gut microbiota produces SCFAs, tryptophan metabolites, and neurotransmitters that exert neuroprotective effects on the brain *via* circulation and the vagus nerve (green arrows). In the state of dysbiosis, TMA/TMAO, pro-inflammatory cytokines, and LPSs are formed, inducing cognitive impairment (red arrows). ECCs, enteroendocrine cells; SCFAs, short-chain fatty acids; LPSs, lipopolysaccharides; TMA, trimethylamine; TMAO, trimethylamine N-oxide. Created with BioRender.com.

The gut microbiota communicates with distant organs and the brain through a complex neuro-humoral connection called the gut–brain axis, which includes: the central nervous system (CNS), the autonomic nervous system, the enteric nervous system (ENS), the hypothalamic–pituitary–adrenal axis, and the immune system. The ENS is the largest part of the peripheral nervous system, consisting of ~200 million neurons and enteric glial cells (the digestive equivalent of brain astrocytes) located along the gastrointestinal tract and often referred to as the “second brain” due to its ability to control gut behavior with and without input from the CNS. Important players in the gut–brain axis are the enteroendocrine cells (EECs). They are specialized cells localized within the intestinal epithelium and represent the sensors of the gut microbiota and its metabolites. In response to luminal content, the EECs secrete hormones and cytokines that can act in a paracrine manner on the ENS or send information *via* the vagus nerve to the CNS (Figure 2). In addition, they are involved in the motility of the gastrointestinal tract, the gut barrier, and mucosal immunity. The vagus nerve, as the main component of the parasympathetic nervous system, establishes one of the connections between the gut, the brain, and inflammation and represents an important link between nutrition and diseases. The CNS affects digestion by regulating gut motility, secretion, and immunity *via* the sympathetic and parasympathetic nervous systems. Neurotransmitters, hormones, and peptides released by the ENS and transported through the bloodstream cross the blood–brain barrier and can act synergistically with the signals sent “down” from the brain through the efferent vagus nerve to regulate food intake and appetite (74). The gut microbiota reacts to these changes by producing neurotransmitters and microbial metabolites, such as short-chain fatty acids (SCFAs), secondary bile acids, and tryptophan- and polyphenol-derived products, which all affect the host's CNS. These processes and connections are schematically presented in Figure 2.

The major metabolites secreted by the colon microbiota, after anaerobic degradation of non-digestible carbohydrates (dietary fibers), are SCFAs: mainly butyric, propionic, and acetic acids (75). Acetate and propionate are produced by the *Bacteroidetes phylum*, while species of the *Firmicutes phylum* preferentially produce butyrate. Different sources of fibers, such as resistant starches (from whole grains and legumes) or fructo-oligosaccharides from bananas, onions, and asparagus, yield different levels of butyrate and other SCFAs. Some prebiotic fibers, such as inulin and fructo-oligosaccharides, promote the growth of commensal bacteria that produce high amounts of butyrate in the gut (76). SCFAs contribute to gut health by regulating the integrity of the intestinal barrier, mucus production, and controlling inflammation by inducing Treg differentiation. Butyrate is used as an energy source by the colonocytes, while the liver clears the majority of propionate and butyrate from the portal circulation (77). However, a minor fraction of colon-derived SCFAs reaches the bloodstream and can be transported to the brain by passing through the blood–brain barrier to exhibit a neuroprotective effect (78). SCFAs are involved in maintaining blood–brain barrier permeability and the CNS immune system by regulating microglial function. The epigenetic effects of butyrate have also been documented, as it is a well-known inhibitor of HDAC, affecting gene expression in the gut and associated immune tissue, as well as in the nervous system. Treatment with sodium butyrate in animal models of Parkinson's disease has been shown to prevent neuronal cell death, while in Alzheimer's disease and traumatic brain injury models memory and learning improved (79). However, chronic, slightly elevated blood propionate and concomitant increased ammonia levels in the circulation may play a role in cognitive impairment and dementia (80).

Gut microbiota species also produce other bioactive compounds, such as folate (vitamin B9), and neurotransmitters, such as serotonin (5-hydroxytryptamine; 5-HT), dopamine, and γ-aminobutyric acid (GABA), as summarized in a recent review (81). The tryptophan microbial metabolite indole also represents an important link between the microbiota and the host, playing a role in the modulation of intestinal epithelial integrity and intestinal inflammation, and it positively correlates with longevity (82). A reduced level of the neurotransmitter serotonin, found in dysbiosis, is related to cognitive impairment (Figure 2).

## Dysbiosis—a Link Between Diet, Aging, and NDD

Dysbiosis is a state of imbalanced abundance and composition in the gut microbiota with changes in microbial-derived products. It often leads to the overgrowth of otherwise low-abundance and/or harmful bacteria. A family of Gram-negative bacteria, *Enterobacteriaceae*, are the most commonly overgrown gut microbes in a wide range of pathologic conditions, including inflammation. Gut inflammation, on the other hand, causes damage to and the death of mucosal epithelium cells. This results in an increase in phospholipids from the membrane lipids of dead cells, which can be used as a source of carbon and/or nitrogen by a variety of species in the *Firmicutes, Actinobacteria*, and *Proteobacteria phyla*, as well as pathogenic species such as *Salmonella* and *Pseudomona*s. An inflamed gut favors the growth of anaerobic bacteria (such as *E. coli*) and mucin-degrading bacteria (*Akkermansia musiniphila* and *B. acidifaciens*), leading to a depletion of commensal bacteria (*Bacteroidetes* and *Clostridia phyla*) and favoring a growth of *Enterobacteriaceae* and pathogens such as *S. Typhimurium* and *Clostridium difficile* (83). Inflammation results in increased intestinal permeability, referred to as “leaky gut,” allowing the translocation of microorganisms and/or their components and metabolites from the gut to the bloodstream. Endotoxins (lipopolysaccharides; LPSs), a major component of the outer membrane of Gram-negative bacteria, after entering the bloodstream and binding with LPS-binding protein (LBP) and CD14 receptor, launch the secretion of pro-inflammatory cytokines. In this way LPSs induce neuroinflammation, which triggers and perpetuates the neurodegenerative process (Figure 2). Although small concentrations of LPSs are detectable in healthy individuals (endotoxemia), elevated postprandial LPS levels after fat-rich meals, referred to as “metabolic endotoxemia,” have been proposed as a major cause of inflammation, including chronic low-grade inflammation (84). Several studies have demonstrated that chronic gastrointestinal syndromes, such as inflammatory bowel syndrome (85), small intestinal bacterial overgrowth (86), and celiac disease, are associated with neurological disease development (87). Dysbiosis has been implicated in worsened outcomes after traumatic brain injury, which has been considered as a non-genetic risk factor for several NDDs, including ALS, AD, and PD (88).

Aging is concomitant with changes in gut physiology, including lower levels of stomach acid and changes in gastric motility and in the ENS, that consequently affect the composition and function of the gut microbiota. The most prominent feature in the microbiota of elderly individuals is a reduced *Firmicutes*-to-*Bacteroidetes* ratio compared with young adults (89) and decreased beneficial *Lactobacillus* and *Bifidobacterium*. Decreased microbial diversity in elderly individuals is associated with increased frailty, blood inflammatory markers, and decreased nutritional diversity. Perturbations in the gut microbiota, such as decreased abundance of bacteria involved in SCFA production and an enrichment of low-abundance pathobionts, further promote and sustain pro-inflammatory conditions (90). Low-grade chronic systemic inflammation accompanied with physiological aging is defined as “inflammaging.” Altered, aged gut microbiota compositions have been proposed to contribute to this heightened pro-inflammatory status characterized with increases in pro-inflammatory cytokines (IL-6 and TNF-α), acute-phase reactants (C-reactive protein), and decreases in IL-10 (91). Inflammaging contributes to the development of age-related diseases: metabolic, cardiovascular, and neurodegenerative (92).

Some recent metabolomic investigations have shown that individual gut microbiomes become increasingly more unique with age, and uniqueness is positively associated with health and longevity. Although, healthy elderly individuals showed a decline in core taxa (dominant genera) and replacement by less common taxa, their gut microbiome continued to show a distinct composition (82). Metabolomic studies correlated three markers of longevity (phenylacetylglutamine—PAG; p-cresol sulfate—PCS; 2-hydroxybenzoate-−2-HB) from the urine of elderly individuals and centenarians with the gut microbiome. PAG and PCS are formed by the microbial catabolism of proteins (phenylalanine and tyrosine metabolites), while 2-HB originates from fruits and vegetables. PAG was positively correlated with *Proteobacteria* species, both PCS and PAG correlated to *Vibrio et. rel*., and 2-HB was found to be positively correlated with *Proteus et. rel*. (93).

Emerging evidence suggests that changes in the function and composition of the gut microbiota contribute to the pathogenesis of NDD by the induction of epigenetic modifications. Epigenetic changes associated with NDD mostly include DNA methylation and histone modifications, which are controlled by several enzymes, such as acetylases and methylases (94). These enzymes are regulated by the metabolites generated by the host's gut microbiota. Such metabolites are short-chain fatty acids, folates, biotin, and trimethylamine-N-oxide (11). The bidirectional interaction between the gut microbiota and epigenetics has been documented, although the nature and significance of this relationship has not been fully elucidated. They may act in synchrony to modulate the pathogenesis and progression of NDD (94, 95). On the other hand, metabolites produced by the gut microbiota may also reverse some of the previously induced epigenetic modifications (96) and thereby prevent the development or progression of NDD.

## Microbiota in Different NDDs

Recent studies have shown decreased intestinal microbial diversity in AD patients, with increased abundance of the *Bacteroides phylum* and decreased abundance in *Firmicutes* (97). Such conditions favored the growth of pro-inflammatory Gram-negative bacteria, such as *Escherichia coli, Shigella, Helicobacter*, and *Odoribacter*, while reducing beneficial commensals such as *Bifidobacterium* and SCFA-producing bacteria (98). This state promotes the formation of amyloid plaques, typically found in AD patients. Another possible mechanism involves bacteria-derived amyloids, produced by *E. coli, Salmonella spp., Pseudomonas fluorescens, Klebsiella pneumonia, Staphylococcus aureus, Bacillus subtilis, Streptomyces coelicolorcan*, that can function as initiators to cross-seed and through molecular mimicry aggregate host amyloids (99). In addition, a chronic *H. pylori* infection could trigger the release of both inflammatory mediators and amyloids in AD patients (100), while the eradication of *H. pylori* has been shown to be associated with a decreased progression of AD symptoms (101). As Aβ plaques have been detected in the intestinal mucosa of both AD animal models and human patients, it is hypothesized that endogenous Aβ production starts in the gut and subsequently spreads to the CNS. Another possible mechanism that causes cognitive deterioration is a release of certain microbial-derived metabolites, such as trimethylamine N-oxide—a product of the metabolic transformation of dietary choline by the gut microbiota to trimethylamine, which is then oxidized by the host's liver (Figure 2) (102). Both trimethylamine and its oxide are involved in aging and neurodegeneration.

Parkinson's disease (PD) is characterized by a loss of movement control due to a decrease in brain dopamine production as a result of the degeneration of substantia nigra neurons. Non-motor symptoms are also present, such as constipation, which may precede the onset of motor deficits years or decades before. A hallmark of PD pathology is an aggregation of misfolded α-synuclein (αSYN), so-called Lewy bodies, in the brain; although it has been found that αSYN aggregates can be present in the ENS of clinically healthy individuals years before they develop PD. Misfolded α-synuclein has also been shown to spread from cell-to-cell and, in a prion-like fashion, from the periphery to the CNS *via* the vagus nerve (103, 104). A large number of studies have shown diversity and abundance in the microbial species present in the gut of PD patients (105). Moreover, a positive correlation between the increased abundance of *Lactobacillaceae* and *Enterobacteraceae* and disease severity has been shown. In PD, observed decreases in the fecal amounts of *Prevotella spp*. and *Clostridium spp*., major producers of SCFAs, folate (vitamin B9), and thiamine (vitamin B1), may have an impact on intestinal epithelial barrier permeability. Furthermore, studies indicate alterations of the bacteriophage community in people with PD (106). Epidemiological data indicate that PD is associated with a variety of enteral dysfunctions: inflammatory bowel disease, *H. pylori* infection, and constipation (105). Recent studies have revealed changes in the gut microbiome as a result of usual PD treatments (107). On the other hand, *Enterococcous faecalis* has been found to metabolize levodopa, a commonly used drug for PD, suggesting that the gut microbiome may reduce the peripheral availability of levodopa and thereby affect the efficacy of the PD treatment (108, 109).

Amyotrophic lateral sclerosis affects the brain and spinal cord neurons, leading to paralysis, respiratory failure, and death. In ALS patients, reduced relative microbial abundances have been found in butyrate-producing *Anaerostipes, Oscillibacter*, and *Lachnospira*, while that of glucose-metabolizing *Dorea* has been found to be significantly increased. ALS patients also have elevated LPS levels in plasma (110), which support neuroinflammation through microglia activation. Multiple sclerosis is an autoimmune NDD, which also appears to be associated with an altered gut microbiota. An increased relative abundance of *Akkermansia* has been shown to be correlated with symptom expression. In addition, MS patients have been shown to exhibit decreased levels of *Parabacteroides distasonis*, a species associated with anti-inflammatory activity (111).

## Precision Nutrition in NDD

Studies with caloric restriction have shown neuroprotective effects in animal models, but the results in humans are inconsistent. However, the Mediterranean diet (112) and the Mediterranean–ketogenic diet (113) improved cognitive function in older people by altering intestinal microbiota and their metabolites.

In addition to the effects on the gut microbiota, diets rich in olives, nuts, or ω-3 PUFA affected genes associated with infection and inflammation. The Mediterranean diet downregulates transcriptional repressor *NFIL3*, which is involved in the regulation of cytokine expression, the development of immune cells, and the circadian clock. Olive oil and nuts have been found to downregulate IL-8, a key mediator of inflammation, and a coagulation factor (SERPINB2) involved in adipose tissue development. Moreover, they downregulate the expression of *RGS1*, a regulator of the G-protein superfamily, which is linked to chronic inflammatory diseases such as celiac disease, MS, and AD. However, the supplementation of a diet with olives, nuts, or ω-3 PUFA dysregulated different genes in AD patients (114).

Numerous nutraceuticals such as curcumin, epigallocatechin-3-gallate, resveratrol, Ginkgo biloba extract, genistein, flavonoids from berries, and polyphenols from extra virgin olive oil and red wine are able to delay neurodegeneration (115). Besides their role in epigenetics, as discussed above, the health benefits of these nutraceuticals in the prevention and alleviation of NDD are due to their ability to change the gut microbiota. Moreover, the bioavailability of polyphenols, PUFAs, and antioxidants with neuroprotective effects depends on the colon microbiota. The differences in gut microbiota composition may explain the inter-individual variability in the outcome of supplementation in clinical trials, emphasizing the need for precision nutrition.

The relationship between microbiota and nutraceuticals is bidirectional. This has been shown for curcumin (116). Curcumin was found to significantly alter the relative abundances of bacterial species, several of which have been associated with the development of AD, while the biotransformation of curcumin by gut bacteria produces neuroprotective metabolites (117). Further, turmeric and curcumin have been shown to exert potential to alter the gut microbiota but with significant variation over time and an individualized response to treatment (118). Flavanols from red wine and cocoa have a positive effect on the gut microbiota by increasing the presence of “healthy” bacteria, such as *Firmicutes* and *Bacteroidetes*, which have been found decreased in cerebral inflammation (119). Probiotic and prebiotic supplementation have shown moderate beneficial effects in AD patients, although oligosaccharides from *Morinda officinalis* (120) and oligomannate from algae (121) exhibit promising effects on animal models of AD. On the other hand, gut-microbiota-derived metabolites from plants may have protective effects against AD, e.g., urolithins, which are metabolites of ellagitannin in, for example, pomegranate fruit (122).

The reversible nature of epigenetic changes has pointed out the specific nutritional interventions aimed at reversing epigenetic modifications to prevent or treat NDD. Although diets rich in bioactive compounds have demonstrated beneficial effects in preventing and modifying NDD, the application of precision nutrition appears to be markedly more effective than the traditional approach. The pathologies of NDD can exert variable clinical characteristics in the patients with the same disease. A combination of genetic and epigenetic factors, lifestyle, and microbiome data could provide a full overview of an individual patient and enable the stratification of patients into specific nutritional groups in order to avoid non-responders to a specific diet and to get a maximum response from the precision nutrition for each patient.

## Conclusions

In summary, NDDs remains an important public health challenge because of the lack of effective prevention or treatment. The reasons for the slow progress in both prevention and therapy may lie in the fact that current recommendations are designed for the general population, without taking into account individual genetic, epigenetic, and lifestyle factors. However, NDDs have complex pathologies with great inter-individual (epi)genetic variance within the population. In addition, the gut microbiome is linked with the development and progression or alleviation of NDDs, through the gut microbiome–brain axis. The relationship between the gut microbiota and epigenetic modifications in NDD is bidirectional and markedly dependent on nutrition. These individual differences in both microbiota and epigenetic signatures suggest the need for personalized dietary plans for NDD patients. A possible target is the creation of personalized dietary interventions containing specific bioactive nutrients that can modify epigenetic changes and/or the gut microbiota. Although numerous studies on the role of different nutraceuticals in the prevention, prediction, and treatment of NDDs have been conducted, we are still far away from a personalized diet plan for individual NDD patients, which is undoubtedly the future of NDD therapy. To achieve this goal as soon as possible, large-scale cohort studies that would include the precise monitoring of food intake, mapping of genetic variants, epigenetic data, microbiome studies, and metabolome, lipidome, and transcriptome data are urgently needed.

## Author Contributions

VV and MM designed the review. MM, AA, and ZC performed the literature analysis and wrote the manuscript. VV critically revised the text and gave a substantial scientific contribution. All authors have approved the final version of the manuscript for publication.

## Conflict of Interest

The authors declare that the research was conducted in the absence of any commercial or financial relationships that could be construed as a potential conflict of interest.

## Publisher's Note

All claims expressed in this article are solely those of the authors and do not necessarily represent those of their affiliated organizations, or those of the publisher, the editors and the reviewers. Any product that may be evaluated in this article, or claim that may be made by its manufacturer, is not guaranteed or endorsed by the publisher.
